# The emerging paradigm in pediatric rheumatology: harnessing the power of artificial intelligence

**DOI:** 10.1007/s00296-024-05661-x

**Published:** 2024-07-16

**Authors:** Oya Koker, Sezgin Sahin, Mehmet Yildiz, Amra Adrovic, Ozgur Kasapcopur

**Affiliations:** 1 Department of Pediatric Rheumatology, Faculty of Medicine, Marmara University, Istanbul, Turkey; 2 Department of Pediatric Rheumatology, Cerrahpasa Faculty of Medicine, Istanbul University-Cerrahpasa, Istanbul, Turkey

**Keywords:** Artificial intelligence, Computer Vision systems, Convolutional neural network, Deep learning, Machine learning, Pediatric rheumatology

## Abstract

Artificial intelligence algorithms, with roots extending into the past but experiencing a resurgence and evolution in recent years due to their superiority over traditional methods and contributions to human capabilities, have begun to make their presence felt in the field of pediatric rheumatology. In the ever-evolving realm of pediatric rheumatology, there have been incremental advancements supported by artificial intelligence in understanding and stratifying diseases, developing biomarkers, refining visual analyses, and facilitating individualized treatment approaches. However, like in many other domains, these strides have yet to gain clinical applicability and validation, and ethical issues remain unresolved. Furthermore, mastering different and novel terminologies appears challenging for clinicians. This review aims to provide a comprehensive overview of the current literature, categorizing algorithms and their applications, thus offering a fresh perspective on the nascent relationship between pediatric rheumatology and artificial intelligence, highlighting both its advancements and constraints.

**Supplementary Information:**

The online version contains supplementary material available at 10.1007/s00296-024-05661-x.

## Introduction

Artificial intelligence (AI) is a sophisticated field of computer science with numerous subfields and a wide array of applications, dedicated to creating systems capable of performing tasks that typically require human intelligence, including learning, reasoning, problem-solving, and understanding natural language [1]. Demonstrating its capabilities in various areas such as literature, art, and creativity, AI is pushing boundaries with its visual and auditory applications and continues to open new horizons in medicine. AI, by mimicking fundamental aspects of a physician and utilizing extensive knowledge repositories, can formulate differential diagnoses, provide preliminary diagnoses, and even make treatment recommendations. In addition to its contributions to clinical practice, it is making strides towards inspiring academics with the increasing research in recent years [2, 3]. It has been demonstrated to be particularly influential in guiding visual assessments and molecular analyses that require standardization and time, especially in areas where traditional scientific methods fall short [3–5]. On the other hand, natural language processing (NLP) models, possessing the ability to comprehend and generate human language, have emerged as one of the most rapidly embraced applications by scholars in the scientific research community, challenging the norms of plagiarism and ethical standards. So, while captivating with their linguistic and analytical capabilities, these applications also spark discussions regarding potential misleading interpretations, simulated citations, and the dimension of academic misconduct [6–8].

Childhood rheumatic diseases are chronic conditions of autoimmune or autoinflammatory origin that affect the joints, muscles, connective tissues, and various other organs. The lack of a diagnostic confirmation test and the necessity for biomarkers, along with the potential importance of imaging or histopathological assessment and staging in treatment decisions for various organ involvements, as well as the significance of autoantibody patterns in autoimmune disorders or genetic analyzes in autoinflammatory diseases, increase the growing expectation for the promising support of AI technology in pediatric rheumatology.

**Fig. 1:**
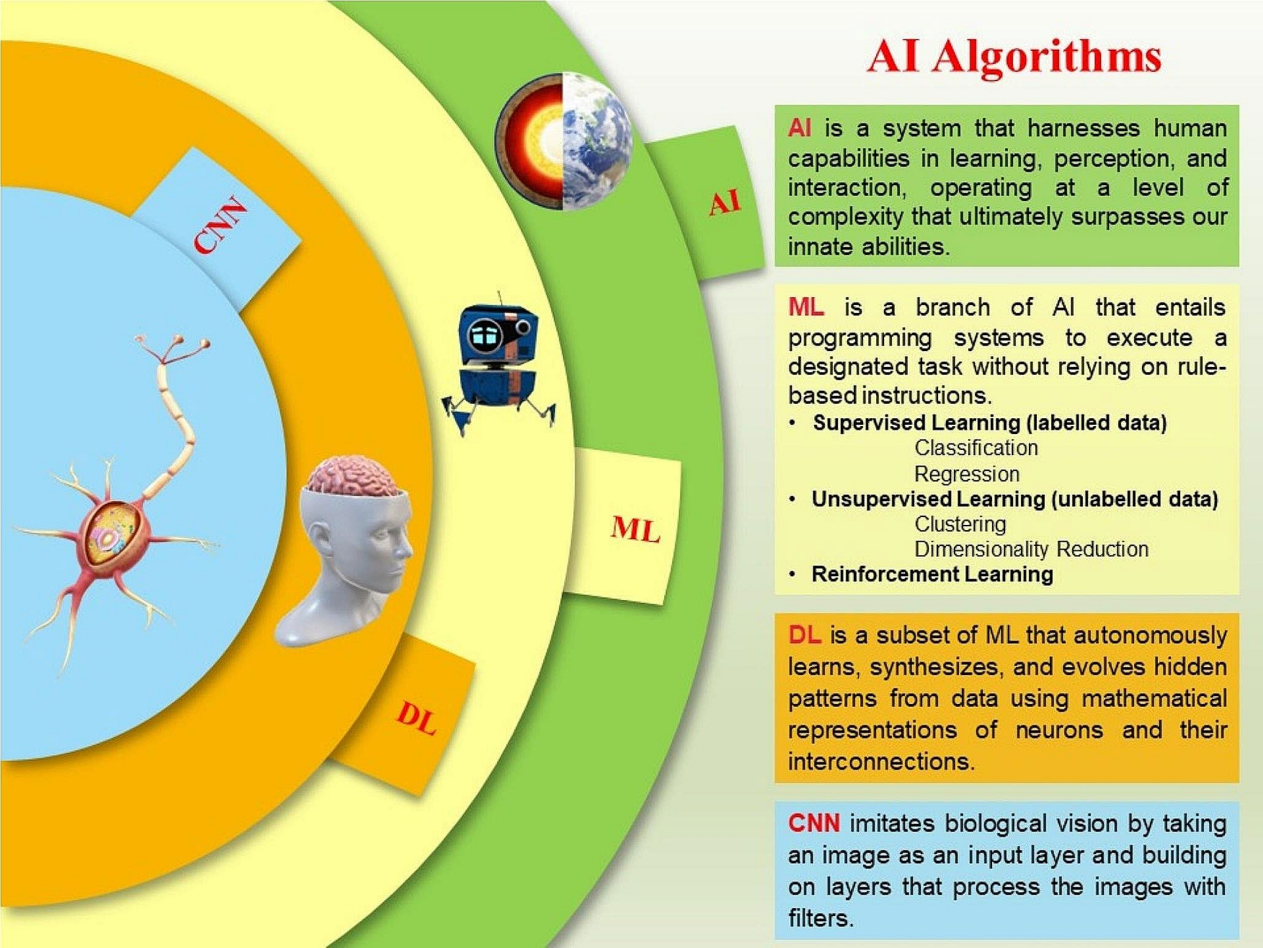
The terminology of artificial intelligence algorithms. (AI: Artificial Intelligence; ML: machine learning; DL: deep learning; CNN: convolutional neural network)

Examining the burgeoning connection between pediatric rheumatology and AI, one notices a noteworthy presence of machine learning (ML) and deep learning (DL) algorithms in a restricted set of studies concerning diagnostic approaches. The enthusiasm surrounding ML stems from a pivotal capability: the potential to analyze intricate and extensive data structures. This ability enables the creation of prediction models tailored to enhance the customization, accuracy, and overall effectiveness of diagnoses, prognoses, monitoring, and treatment administration and ultimately improve individual health outcomes [9–12]. Diverging from traditional methods, it adeptly assesses potential connections between variables without adhering to fixed assumptions and hypotheses, skillfully transcending established patterns [13, 14]. The terminology of AI algorithms is depicted in Fig. 1. This review aims to provide a comprehensive overview by evaluating the existing literature on the recent relationship between pediatric rheumatology and AI.

## Search methodology

Original research articles discussing the application of AI in pediatric rheumatology and review articles addressing the relationship between rheumatology and/or pediatric rheumatology and AI have been examined within the scope of the subject. The research and methodological approach were informed by previously published scholarly works [15, 16]. Two authors (OK and SS) independently conducted the literature review. Consistency and appropriateness were verified and confirmed by another author (OK) and reconciled by consensus. An electronic literature search was performed, covering articles from the inception of the databases to May 2024. Boolean operators and medical subject heading terms were used to enhance electronic searches. To systematically address the topic and grasp the current classification and terminology, the search terms “artificial intelligence”, “machine learning”, “deep learning”, “big data”, “supervised learning”, “unsupervised learning”, “reinforcement learning”, computer neural network, “convolutional neural network”, “musculoskeletal”, “rheumatology”, and “pediatric rheumatology” were utilized in searches conducted through Medline/PubMed, Scopus, Web of Science, and Directory of Open Access Journals (DOAJ). Further searches were performed on references of the included studies to ensure comprehensive coverage.

## Selection criteria

The titles and abstracts of all articles relevant to the subject have been evaluated, and the eligibility criteria have been determined by all authors. Articles published in English have been considered. Publications that were not full-text articles (e.g., conference, abstract) have been excluded. The authors have re-analyzed the full texts of review articles exploring the relationship between rheumatology and AI, as well as studies that employed AI algorithms in their methodologies. Data extraction has focused on AI methods used, and outcomes related to pediatric rheumatology. The final reference list was compiled based on the originality and relevance to the broad scope of this review. Finally, the data and information obtained from the publications were systematically categorized and transcribed in line with the main themes and objectives, the AI subtypes and algorithms, the results, and the limitations. Additionally, review articles relevant to the topic were utilized in the description and classification of the methods.

**Fig. 2:**
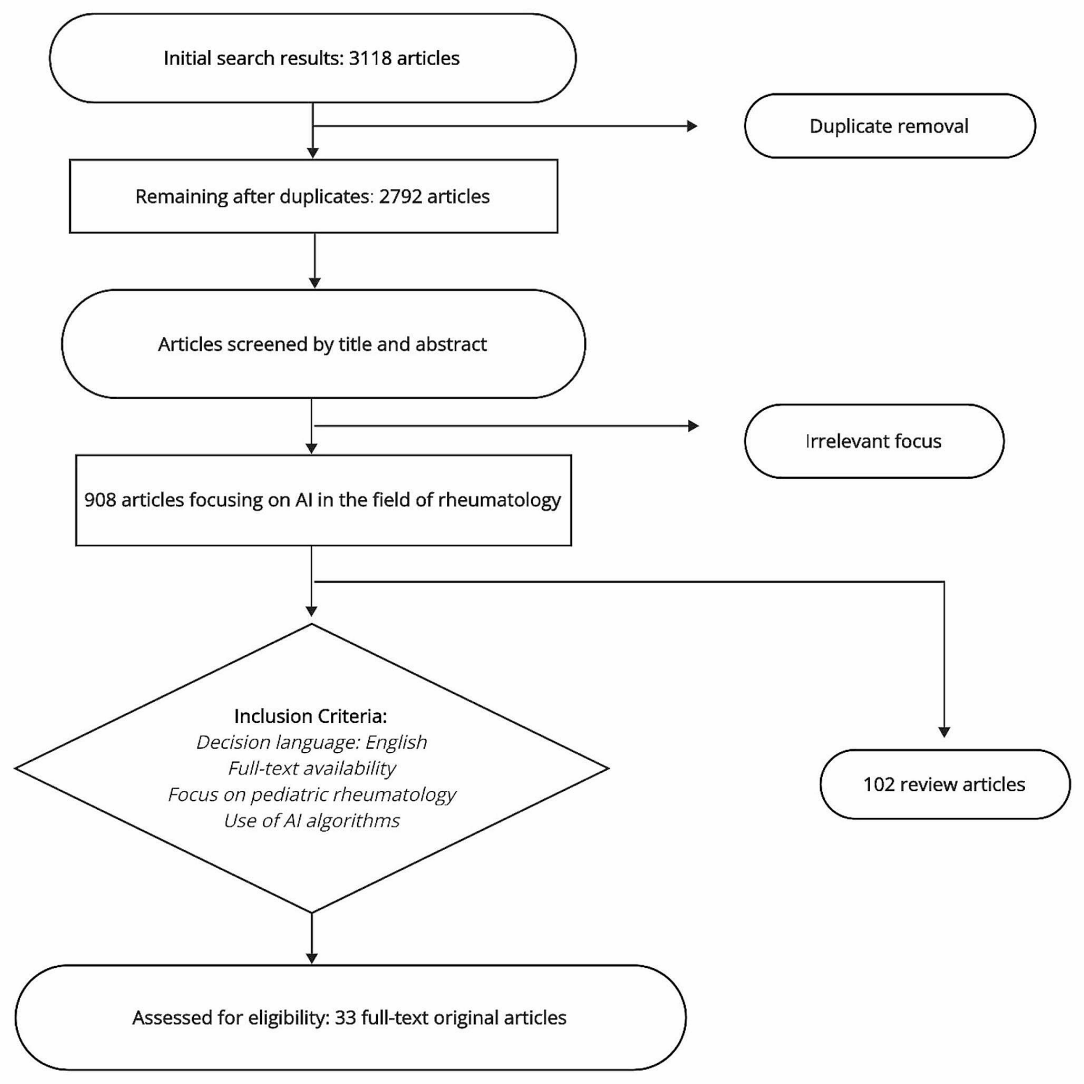
Flow diagram reflecting the search methodology of the review

To ensure standardization, AI algorithms were examined and categorized into four main subtypes (ML, DL, NLP, expert systems), each with its own subcategories, and recorded accordingly [4, 17]. ML was evaluated under the subheadings of supervised learning, unsupervised learning, and reinforcement learning. Within these subheadings, classification algorithms (such as logistic regression, Support Vector Machines (SVM), decision trees, random forest (RF), k-Nearest Neighbors (k-NN), and Naive Bayes) and regression algorithms (Linear Regression, Polynomial Regression, Ridge Regression, and Lasso Regression) were considered in line with the methodologies of the research articles. Clustering algorithms such as k-Means and hierarchical clustering were also included. Neural networks were assessed under the DL subheading, while Rule-Based, knowledge-based, and fuzzy logic systems were categorized under expert systems. A flow diagram presenting the search strategy and steps is depicted in Fig. 2.

## Artificial intelligence algorithms in pediatric rheumatology

### Knowledge-based systems

As computer systems gained the ability to think and learn, the concept of ML, a subset of AI, emerged. Rooted in the 1940s, this concept has advanced in recent years due to the digitization of extensive datasets, the development of high-capacity general and graphics processors with superior analytical capabilities and minimal error rates, and its capacity to provide open access to information [18, 19]. The technology we are immersed in daily through smartphones and computers has become a subject of discussion in pediatric rheumatology literature due to its ease in diagnosis, monitoring, and communication. In the 1980s, expert systems mimicking a clinician’s diagnostic reasoning, consisting of a knowledge base and an inference mechanism component, were introduced [20–22].

A knowledge-based system takes patient data as input and uses an inference engine to match that data with the repository of expert knowledge, providing diagnostic recommendations or treatment options [23, 24]. These systems can assist clinicians by providing evidence-based recommendations and identifying patterns in complex clinical data. The computer-based consultation system AI/RHEUM has been developed for non-rheumatologist physicians to provide diagnostic assistance in this specialty for adults. Although described as promising systems with moderate to excellent performance, none have successfully managed to integrate into daily practice [23]. When evaluated for diagnosing pediatric rheumatic diseases, a modified version of this diagnostic decision support system has achieved a diagnostic accuracy of 92% [24]. This system has been found to be instructive by experts. On the other hand, it has been emphasized that before the more widespread introduction of such expert systems, assessing the sensitivity of the system in diagnosing conditions, reviewing the legal aspects, and resolving financial issues are essential. Recently, an AI-based system named *Juvenile Idiopathic Arthritis Dialogue-based Education* (JADE) has been developed through a dialogue system to meet the educational needs of families of children with Juvenile Idiopathic Arthritis (JIA) [25]. This system, which contains fundamental information about JIA and generates interactive dialogues that allow parents to ask questions, has aimed to inform parents about JIA better and address their inquiries. The results have been successful; however, concerns about bias arose due to the participants being from a single region and the analysis being conducted by a single coder. Additionally, the limitations have included awareness and experience requirements in using the system [25].

As AI continues to advance, applications that enhance communication also emerge. Large language models and AI-powered chatbots have the potential to provide significant support to families of children with chronic rheumatic conditions, addressing the psychosocial aspects of these diseases. In the United Kingdom, a co-designed proof-of-concept study has been planned to design, develop, and test a chatbot intervention [26]. These chatbots aim to help parents manage their children’s conditions more effectively between hospital visits. By addressing gaps in current clinical care and incorporating user feedback, such AI interventions can enhance the overall well-being of young patients and their families, potentially leading to broader implementation and efficacy trials in the future. In a recent study from a different geographic region, attention was drawn to Generative Pre-trained Transformer (ChatGPT) ‘s ability to process complex information and perform scientific reasoning with sensitivity [27]. Yet, it was also emphasized that caution is necessary as its outputs are inferences from the input literature and may be detrimental to clinical practice.

The guidance of patients through digital symptom assessment tools holds promise, particularly in addressing communication and transportation barriers to some extent and expediting the diagnostic process. Comparisons between the diagnostic accuracy of experienced rheumatologists and that of an AI-based symptom monitoring device have yielded encouraging results [28]. However, the integration of these tools into pediatric practice, which requires more detailed examinations and meticulous differential diagnoses, appears challenging.

### Prediction models for diagnosis and outcome

Laboratory use in rheumatic diseases guides the diagnostic approach, but it is not conclusive. In the presentation of the disease, certain clinical findings and values can serve as signals for prognosis and shape treatment management. In a study aiming to create opportunities for conservative approaches in Multisystem inflammatory syndrome in children (MIS-C) management, differentiation of risk groups was intended, and the prognostic values of key clinical and laboratory features in the disease were evaluated via classic ML algorithms (decision tree, SVM, logistic regression analysis, naïve Bayes, and linear discriminant analysis). It has been concluded that brain natriuretic peptide, total protein, ferritin, and D-dimer laboratory tests demonstrated the highest performance [29]. On the other hand, the clinical utility of certain indicators that are essential for differential diagnosis or prognosis may be limited in practical clinical use. Thus, another study aimed to develop a model for screening potential patients diagnosed with anti-melanoma differentiation-associated protein 5 (MDA5) antibody-positive juvenile dermatomyositis (JDM), taking into consideration the limited access to myositis-specific antibodies in many developing countries due to financial and technological challenges. The final prediction model, incorporating eight clinical variables and four auxiliary results, yielded a high predictive accuracy for the risk of anti-MDA5 antibody in JDM, demonstrated by an AUC of 0.975 and validated internally with robust metrics, suggesting its superiority over traditional logistic regression models. In addition to the necessity of external validation to demonstrate the accuracy of this model under different conditions, the value of screening for these antibodies in the proposed group has been emphasized [30]. In another study, a nomogram has been constructed utilizing non-invasive clinical features from patients diagnosed with JDM (sedimentation, interleukin-10, MDA-5). The study concluded that, despite certain methodological limitations, this prediction model could offer clinical guidance in evaluating secondary interstitial lung disease in JDM and predicting long-term prognosis [31].

In the saliva of children with Sjögren’s syndrome (*n* = 16), 105 chemokines, cytokines, and biomarkers (CCBM) have been identified 43 of which have exhibited differences compared to healthy controls (*n* = 11) [32]. ML methods have been employed to assess the predictive power that links these CCBMs to the disease. It has been highlighted that further studies are needed to determine whether the newly identified CCBMs in the saliva of children diagnosed with Sjögren’s syndrome are reliable early indicators of the disease or rather a representative of the pediatric-specific disease process [32, 33].

### Real time data and biomarkers

Another area of application is the development of machine-assisted tangible tools as biomarkers, allowing real-time activity monitoring. For instance, a non-invasive and practical tool that assesses knee involvement and can detect activity has been suggested as a biomarker in JIA. Documenting joint acoustic emissions bilaterally and applying signal changes to a ML algorithm has allowed for differentiating children diagnosed with JIA from healthy controls [34]. It has been asserted that this tool could enable screening, monitoring, and prompt treatment. On top of that, the concept has emerged that wearable devices with digital biomarkers could serve as future tools for home disease monitoring, providing objective data for disease tracking [11, 35]. Thermal imaging, capable of detecting temperature alterations in tissue abnormalities, has been explored in the recognition of inflammation in knee and ankle joints in JIA. Despite observed correlations between thermal and visual imaging data, conflicting results have been obtained in different studies [36, 37]. Accelerometers and gyroscopes, capable of measuring the degree of restricted motion in the affected joint by comparing it with normal reference values or the unaffected joint, have been developed [38]. In adult studies, the use of acceleration patterns has been examined to distinguish differences between osteoarthritis, rheumatoid arthritis, and spondyloarthritis [39, 40]. Consequently, the hypothesis has been proposed that these acceleration patterns could also be employed to objectively assess affected joints in JIA [41].

### Stratifying patient cluster

The inadequacy of the proposed classification criteria to encompass all patients, and the heterogeneity in disease progression and treatment response have necessitated the asset for genomic technologies to formulate a classification that reflects both the clinical phenotype range and the underlying biology of JIA. In 2009, it was demonstrated that previously unidentified cytokines could be determined through cluster analysis of multiplex data, revealing that systemic JIA (sJIA) has a different profile compared to oligoarticular and polyarticular JIA [42]. Furthermore, Van Nieuwenhove et al. aimed to identify common immune signatures between subtypes using the RF technique. The immune signature was particularly prominent in active patients and systemic types. Additionally, the ML analysis of the dataset was able to distinguish patients with JIA from healthy controls with an accuracy of approximately 90% [43].

In the last decade, the use of SVMs along with gene expression profiles in peripheral blood mononuclear cells (PBMCs) has come to the forefront in various medical fields. Applying transcriptomic techniques such as microarray or sequencing to the blood or synovial fluid of rheumatic patients holds promise for disease definition and outcome prediction. While it has been demonstrated that the gene expression signatures in PBMCs from polyarticular JIA patients reflect distinct disease processes and provide a molecular classification of the disease, the goal was to further develop this idea with the support of ML [44, 45]. A relatively ethnically heterogeneous JIA cohort consisting of 23 patients in remission and 27 patients with active disease was planned to be stratified by disease activity with segregated PBMC transcriptomes [46]. For this purpose, four common algorithms, including kNN, RF, SVM with cubic kernel (cSVM), and SVM with Gaussian kernel (gSVM) have been considered. After developing models on the entire dataset for predicting the disease stage, efforts have been made to determine whether the inclusion of different patient populations would impact the model’s performance. The results supported the notion that PBMCs, with the use of fitting analytical tools to enhance classification algorithms, constitute a promising source for developing expression-based biomarkers [46]. Similarly, in another recent study, transcriptome data from whole blood gene expressions have been examined aiming to differentiate rheumatic diseases from reactive/infectious conditions. Using the RF algorithm, it has been emphasized that variations in gene expression in blood cells might precede clinical symptoms. The conclusion drawn was that this observation could be beneficial in identifying new biomarkers for pediatric rheumatic diseases [47]. On the other hand, the heterogeneity within each subset, including a limited number of samples, highlights the need for further validation.

In another study utilizing transcriptomic data, a diagnostic model based on the RF algorithm has been developed as a way to distinguish between children with sJIA and healthy children [48]. Through an in-depth examination of datasets from public genetic databases, this study identified four key genes (ALDH1A1, CEACAM1, YBX3, and SLC6A8) that could serve as crucial biomarkers for sJIA. By employing RF techniques with a composite panel of clinical and biomarker variables in non-sJIA patients, the authors have observed enhanced prediction of inactive disease after 18 months, surpassing the predictive capability of conventional determinants alone. The statement suggests that if validated in external cohorts, this approach could pave the way for more rationally designed, biologically based, and personalized treatment strategies in early JIA [48].

In a study utilizing clinical and cytokine expression data, probabilistic principal components analysis (PPCA) through cluster analysis has been employed to identify homogeneous disease subsets [49]. In another study by the same group, sparse multilayer non-negative matrix factorization (NMF) has been developed to uncover data-driven joint patterns predicting clinical phenotypes and disease course. Seven distinct patterns were identified among clinical subtypes [50]. These studies demonstrate that unsupervised ML can identify clinically and biologically significant patterns and classifications. As a result, a stronger connection will be established between clinical outcomes and treatment response, providing evidence that guides the management process.

### Visual analyses

The concept of DL, a subset of ML, undoubtedly plays a significant role in the revival and evolution of AI. DL, manifested through machine perception and computer vision methods, is used in the analysis of medical images with high sensitivity, specificity, and accuracy. AI techniques may help diminish dimensionality or recognize patterns that are not noticeable to the human eye and brain with its detection, quantification, and classification tasks [51, 52].

Most of the studies integrating DL algorithms in rheumatology have been conducted on small and homogeneous datasets [11]. Looking at the current literature in the field of pediatric rheumatology; in a study evaluating the retrospective treatment response of patients diagnosed with Chronic Non-Bacterial Osteomyelitis (CNO), the aim was to develop an ML algorithm that could compare whole-body magnetic resonance imaging (WBMRI) images before and after pamidronate treatment. The results of this algorithm were then intended to be compared with the analysis of a panel of pediatric radiologists [53]. As a result, while machine algorithm could detect new lesions or resolution of a lesion with good precision, it had been unable to accurately classify stable disease [53]. Consequently, the authors underscore the importance of additional research to validate the model in a prospective manner in real-time and ascertain its practicality in a clinical environment.

In another study using visual samples, the potential of AI to discriminate children with JDM from healthy controls and assess the capability of nailfold capillaroscopy (NFC) images to reflect disease activity have been evaluated. The assessment of 1120 images obtained from 111 patients and 321 images from 31 healthy controls resulted in the conclusion that a deep neural network named NFC-Net provides a reliable indicator for discrimination and disease status [54].

### Individualized treatment algorithms

Another agenda route is the elucidation of patients’ clinical and genetic characteristics through AI technology, aiming to create personalized treatment algorithms. In two separate studies conducted by the same group, ML-based models have been developed using electronic medical records to predict the efficacy of methotrexate and etanercept earlier and accurately in JIA patients [55, 56]. The goal was to provide convincing evidence and guidance for treatment algorithms. Among the models used in comparing the results, Extreme Gradient Boosting (XGBoost) algorithm, which works on decision trees, seems to stand out in terms of effectiveness with an accuracy rate of 94.52%. In a different study, the same prediction model was used to predict kidney damage in children diagnosed with IgA vasculitis based on clinical data, demonstrating its potential to reduce the negative effects of invasive procedures [57]. The revealed methods may provide insights into the prognosis and potential complications of the disease, guiding the development of individualized approaches and algorithms.

Another goal with the use of advanced algorithms is to contribute to a better understanding of molecular mechanisms and the identification of advanced treatment strategies through silico models based on systems biology. A target-specific treatment strategy for Still’s disease has been investigated using the therapeutic performance mapping system (TPMS), which relies on pattern recognition techniques to create mathematical models simulating the pathophysiology of humans in silico by integrating existing biological, pharmacological, and medical knowledge [58]. The results have confirmed the use of biologics as a suitable immunomodulatory treatment strategy for Still’s disease and supported the benefits of early IL-1 blockade [59]. However, the precise time for windows of opportunity has not been determined for these interventions.

In a study from the United Kingdom, the response to methotrexate treatment was assessed using AI methods, resulting in the identification of six different patterns (prediction model AUC values 0.65–0.71). Consequently, beyond traditional yes/no assessments (e.g., ACRPedi30), clusters differing by time or individually have been obtained [60]. Advancements in rheumatology healthcare are underway through the utilization of digital technology, leveraging real-world data and evidence. This involves detecting minimal changes in the disease process, monitoring adverse effects and effectiveness of treatment, and enhancing therapeutic efficacy [61, 62].

### Focusing on the advancements and limitations

It seems apparent that AI technologies will streamline access to data and enhance efficiency in the realm of pediatric rheumatology, a domain still in the process of maturation. It can provide diagnostic awareness and enable early diagnosis, treatment, data sharing, and communication. On the other hand, efforts to develop biomarkers, which are currently lacking in detecting and monitoring disease activity and treatment response, are precious. Potential complications can be thwarted thanks to tools that will allow nigher and real-time monitoring of disease activity and enable timely intervention [62]. In clinical research, the development of targeted and individualized treatment approaches through multifaceted analysis of large data sets is seen as one of the main goals [10, 11, 52, 63].

Studies focusing on the application of AI in pediatric rheumatic diseases have been designed with the support of biomedical and computer sciences, and they are quite limited in number. The prominent limitations in the current literature are the inadequacy of data breadth and diversity. Just as seeing a substantial number and variety of patients is important for a physician to gain experience, having a wide range of input-output relationships is crucial for a machine to reduce bias and error rates. In this respect, standard protocols encouraging database sharing among different clinical centers can be developed. Additionally, creating representative training datasets that embrace diverse ethnic and geographic compositions, approaches, and procedures is crucial for clinical applicability and reliability [64]. Another issue is the inability to achieve a balance of fit, leading the model to exhibit overfitting or underfitting. Ensuring a balance between model complexity and the training process can aid in generating effective predictions [13, 64]. Legal and ethical concerns persist, particularly regarding the accountability of clinical decisions made by machines and the accumulation of substantial volumes of sensitive data [65]. Furthermore, research involving children requires strict ethical considerations to protect their physical and psychological well-being. Since children typically cannot provide informed consent, consent must be obtained from their parents or legal guardians. Additionally, it is crucial to ensure that healthcare professionals, parents, and patients are adequately informed and educated about the procedure. When dealing with rheumatology registries, it is pivotal to address bioethical issues by meticulously considering privacy provisions, establishing strict ethical guidelines and ordinances, and ensuring their alignment with the pertinent national and regional legal frameworks [66, 67]. Details and limitations of existing literature using ML algorithms with different goals are presented in Table 1.

**Table 1:** Details of current literature utilizing machine learning algorithms

Publication	Target	Methods	Results	Limitations
Xue et al. [30]	to develop a prediction model for screening anti-MDA5 in JDM	logistic regression, LASSO and RF methods	AUC = 0.97, the final prediction model with eight clinical variables and four auxiliary results	lack of external validation, concerns with applicability
Hu et al. [31]	to establish a prediction model for JDM-ILD by analyzing non-invasive clinical characteristics	SVM	ESIM model including ESR, MDA-5, and IL-10, discriminative in the discovery cohort AUC = 0.736; the validation cohort AUC = 0.792	insufficient breadth of data, lack of external validation
Gomez Hernandez et al. [32]	to assess the presence of CCBMs in the saliva of children with SS	k-NN classifier	AUC = 0.93	lack of external validation
Goossens et al. [34]	to evaluate the use of JAEs as a biomarker to identify knee involvement in JIA	XGBoost classifier,	AUC = 0.81	need for more objective labeling methods and longitudinal monitoring to enhance the results
Van Nieuwenhove et al. [43]	to identify immune signatures defining JIA and discriminate the subtypes	RF technique	AUC = 0.89	insufficient breadth of data, lack of external validation
Poppenberg et al. [46]	to predict disease stage in JIA	SVM, RF, k-NN	accuracies for training > 74% AUC = 0.84 for testing > 78% AUC = 0.94	insufficient diversity and breadth of data, lack of external validation
Eng et al. [50]	to identify patterns of joint involvement and predict the course of JIA	unsupervised ML-NMF		insufficient diversity and breadth of data
Mo et al. [55]	to predict the efficacy of MTX treatment in JIA	XGBoost, SVM, RF	sensitivity 95.35%, specificity 93.33%, accuracy 94.35% AUC = 0.99	small sample size, inadequate representative data, retrospective design
Bhat et al. [53]	to compare segments of pre- and post-pamidronate images of WBMRI in CNO	unsupervised method for clustering and supervised ML-SVM for classification and regression	AUC for Class I- improved was 0.89, Class R-regressed was 0.91 and Class S-stable was 0.68.	insufficient diversity and breadth of data, lack of external validation, concerns with applicability, single encoder, non-representative data

*anti-MD5: Anti-melanoma differentiation-associated protein 5; JDM: Juvenile dermatomyositis; LASSO: Least Absolute Shrinkage and Selection Operator; RF: Random Forest; AUC: Area under the curve; ILD: Interstitial lung disease*,* SVM: Support vector machine; CCBMs: Chemokines*,* cytokines and biomarkers; SS: Sjogren’s syndrome; k-NN: k-Nearest Neighbour; JAEs: Joint acoustic emissions; XGBosst: Extreme Gradient Boosting; JIA: Juvenile Idiopathic Arthritis; ML: Machine learning*,* NMF: Non-negative matrix factorization; MTX: Methotrexate; WBMRI: Whole body magnetic resonance imaging*,* CNO: Chronic nonbacterial osteomyelitis*.

This review has several limitations. The primary limitation is the diversity of AI algorithms and, consequently, the variation in study methodologies, which creates challenges in reaching standardized and definitive conclusions. Additionally, divergences in terminology and classification can impact the number of articles retrieved. However, in this review, along with commonly used standardized terms, specific keywords were employed to ensure comprehensive coverage.

## Conclusions

Artificial intelligence has the potential to enhance ground truth by improving sensitivity, specificity, repeatability, time efficiency, and cost-effectiveness in a specific evaluation [13, 51]. There is a need for AI technologies in the field of pediatric rheumatology. Without ignoring ethical concerns regarding data privacy, the limiting factors of the existing literature should be addressed, and the focus should be on exploring various strategies to overcome them. Technical support and expertise will be required in the development of DL algorithms, but the clinical experiences and knowledge of rheumatologists will shed light on these studies. Therefore, interdisciplinary teamwork is required with the close collaboration of clinicians, biomedical informatics scientists, and ML experts. There is a need for guidelines and general advice on how to ensure and advance the management of big data in a collaborative and ethical manner [13, 67, 68].

## Electronic supplementary material

Below is the link to the electronic supplementary material.

Supplementary Material 1
